# Identification of MicroRNAs as Breast Cancer Prognosis Markers through the Cancer Genome Atlas

**DOI:** 10.1371/journal.pone.0168284

**Published:** 2016-12-13

**Authors:** Jeremy T-H. Chang, Fan Wang, William Chapin, R. Stephanie Huang

**Affiliations:** 1 Biological Sciences Collegiate Division, University of Chicago, Chicago, Illinois, United States of America; 2 Department of Medicine, University of Chicago, Chicago, Illinois, United States of America; 3 Pritzker School of Medicine, University of Chicago, Chicago, Illinois, United States of America; National Institute of Technology Rourkela, INDIA

## Abstract

Breast cancer is the second-most common cancer and second-leading cause of cancer mortality in American women. The dysregulation of microRNAs (miRNAs) plays a key role in almost all cancers, including breast cancer. We comprehensively analyzed miRNA expression, global gene expression, and patient survival from the Cancer Genomes Atlas (TCGA) to identify clinically relevant miRNAs and their potential gene targets in breast tumors. In our analysis, we found that increased expression of 12 mature miRNAs—hsa-miR-320a, hsa-miR-361-5p, hsa-miR-103a-3p, hsa-miR-21-5p, hsa-miR-374b-5p, hsa-miR-140-3p, hsa-miR-25-3p, hsa-miR-651-5p, hsa-miR-200c-3p, hsa-miR-30a-5p, hsa-miR-30c-5p, and hsa-let-7i-5p —each predicted improved breast cancer survival. Of the 12 miRNAs, miR-320a, miR-361-5p, miR-21-5p, miR-103a-3p were selected for further analysis. By correlating global gene expression with miRNA expression and then employing miRNA target prediction analysis, we suggest that the four miRNAs may exert protective phenotypes by targeting breast oncogenes that contribute to patient survival. We propose that miR-320a targets the survival-associated genes *RAD51*, *RRP1B*, and *TDG*; miR-361-5p targets *ARCN1*; and miR-21-5p targets *MSH2*, *RMND5A*, *STAG2*, and *UBE2D3*. The results of our stringent bioinformatics approach for identifying clinically relevant miRNAs and their targets indicate that miR-320a, miR-361-5p, and miR-21-5p may contribute to breast cancer survival.

## Introduction

Breast cancer is the second-most common cancer and second-leading cause of cancer mortality in American women. [1] The number of new breast cancer cases is estimated to reach 249,260, and the number of deaths related to breast cancer is estimated to surpass 40,000 in the United States for 2016. [2] Challenges facing the effective management and treatment of breast cancer include chemoresistance as well as distant site metastasis, which is the leading cause of death within breast cancer cases. [3,4] Increasing our understanding of breast tumor biology can lead to the development of improved diagnostic and prognostic tools, as well as more efficacious therapies for breast cancer.

MicroRNAs (miRNAs) are 22–25 nucleotide RNA segments that engage in post-transcriptional regulation by targeting messenger RNA sequences. [5] The regulatory network of specific genes and miRNAs is often multifaceted because multiple miRNAs may regulate the same gene while at the same time a single miRNA can target multiple genes. [6] Given the regulatory abilities of miRNAs in processes such as cell proliferation, adhesion, and migration, the dysregulation of miRNAs has proven to play a significant role in cancer, including breast cancer. [7–9] The role of miRNAs in breast cancer presents a promising approach for better comprehending breast cancer development, chemoresistance, and metastasis.

In recent years, the use of data-mining and bioinformatics for genomics analyses has increased immensely due to the introduction of new technologies and large-scale efforts to construct useful databases. The Cancer Genome Atlas (TCGA) represents one of the largest collections of genomic data for breast cancer, possessing both clinical and molecular information for over 1000 breast cancer cases. [10] The goals of this study were to implement a bioinformatics approach combining clinical and molecular data to identify miRNAs with prognostic value and to explore the potential gene targets of these miRNAs.

## Materials and Methods

### Identify miRNAs whose expression is correlated with breast cancer survival

From the breast cancer project of TCGA, the miRNA (Illumina) HiSeq dataset and overall survival dataset were downloaded from TCGA data portal. The miRNA HiSeq dataset contained 759 patient samples. Only those mature miRNAs that had expression values in >90% of samples were further evaluated. This led to a subsequent analysis of 309 mature miRNAs. Both a generalized linear model (glm) and a linear model (lm) analysis were performed between the miRNA HiSeq dataset and overall survival using the coding language R.

**Fig 1:**
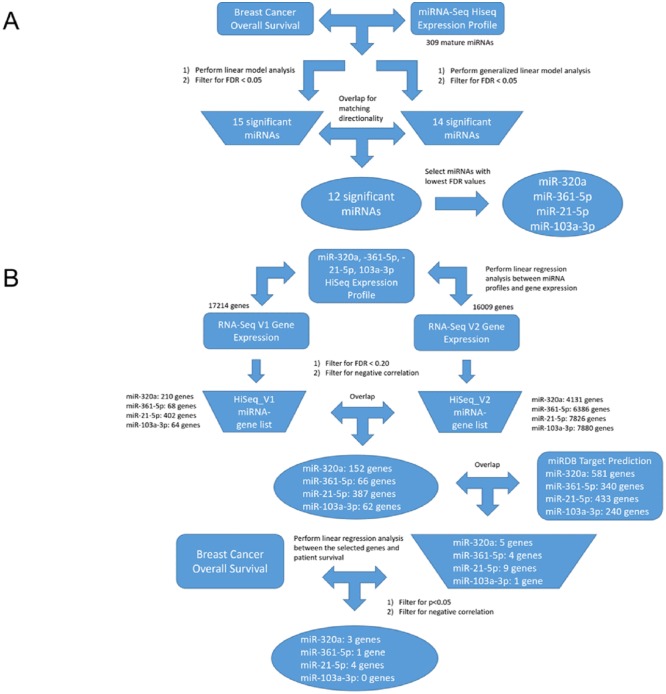
Selection pipeline for miRNAs of interest and finding miRNA-gene interactions in TCGA. A) Selection pipeline to identify clinically relevant miRNAs in breast cancer. Expression data for miRNA and overall survival data (rectangular shapes) were obtained from TCGA breast cancer dataset. Intermediate lists (trapezoid shapes) and findings (oval shapes) derived from our TCGA analysis. B) Selection pipeline to discover possible miRNA-gene interactions. Data retrieved from other sources (rectangular shapes) were obtained from TCGA or miRDB. Intermediate lists (trapezoid shapes) and findings (oval shapes) derived from our analysis.

The miRNAs with FDR<0.05 and those possessing matching directionalities to survival in both the glm and lm analyses were overlapped. Finally, the miRNAs with the lowest FDR values in both analyses were chosen as our miRNAs of interest. The overall selection pipeline for identifying the miRNAs of interest is outlined in Fig 1A.

The Kaplan—Meier method and log-rank test was also applied to the breast cancer TCGA data to further assess the clinical relevance of the four miRNAs of interest. Comparisons between patients with low and high miRNA expression were made by separating patients into either the low expression group (n = 379) or high expression group (n = 380) based on the median value of the miRNA’s expression. Survival analysis was performed using GraphPad Prism (Prism Software Corp., Irvine, CA). Log-rank P < 0.05 was considered statistically significant.

### Identify potential gene targets of miR-320a, miR-361-5p, miR-21-5p and miR-103a-3p through TCGA breast cancer gene expression datasets

Two gene expression profiles—RNA-Seq HiSeq V1 and V2—were downloaded from TCGA data portal. The two RNA-Seq datasets were investigated for batch effects with the TCGA Batch Effects Tool developed by MD Anderson Cancer (http://bioinformatics.mdanderson.org/tcgabatcheffects), which utilizes batch IDs to generate the Dispersion Separability Criterion (DSC); higher DSC values indicate greater dispersion between batches than within batches while lower values indicate weak batch effects. The V1 dataset contained gene expression profiles for 881 patients while the V2 dataset contained gene expression profiles for 1215 patients. The two datasets represented two versions of gene expression information from the same sample pool, in which the algorithms for measuring relative gene expression were different. Given the V1 and V2 datasets were both valid expression datasets, we analyzed the two separately against the miRNAs of interest. Once again, when applying a filter for genes that contain values in >90% of samples, the V1 dataset retained 17214 genes while the V2 dataset retained 16009 genes for analysis. The pipeline for finding the gene targets of the miRNA is shown in Fig 1B.

We performed linear regressions between each of the four miRNAs of interest and the gene expression datasets. In other words, each miRNA was analyzed in relation to the V1 dataset and the V2 dataset independently. The results for each miRNA-gene relationship were then filtered by FDR<0.20 and for negative correlations because miRNAs traditionally exert negative regulation on direct gene targets. An overlap of between the separate V1 and V2 analyses for each of our miRNAs of interest produced a list of potential miRNA-gene relationships.

### Predict the compatibility of miRNAs with the selected genes and confirm the oncogenic roles of these genes

The miRNA-gene relationships were validated by testing the compatibility between the miRNA sequences and gene sequences using the miRNA target prediction tool miRDB. [11, 12] The lists of predicted gene targets for the miRNAs were then overlapped with the genes that were produced from the correlation results from TCGA data to find the potential regulatory networks of the four breast cancer-associated miRNAs. Lastly, the gene targets produced from the overlap were analyzed for their correlation to survival by a linear regression. The miRNA-gene interactions that were found to be clinically relevant were then further confirmed by utilizing additional miRNA target prediction tools such as TargetScan (http://www.targetscan.org/vert_71/) and DIANA (http://diana.imis.athena-innovation.gr/DianaTools/index.php). Both the HiSeq V1 and V2 gene expression profiles were evaluated in relation to breast cancer patient survival.

## Results

### Analysis of TCGA miRNA expression identified miRNAs associated with breast cancer survival

To identify clinically relevant miRNAs in breast cancer, the miRNA HiSeq profile was downloaded from TCGA and a correlation analysis was performed (using both the glm and lm function of R) with breast cancer survival. In the glm analysis, the expression of 14 mature miRNAs were correlated with survival (FDR<0.05). The lm analysis revealed 15 such miRNAs (FDR<0.05). The directionality of the two lists of miRNAs were compared and overlapped to yield 12 common miRNAs (Table 1). All 12 of the miRNAs were positively correlated to survival, meaning that higher expression of the miRNAs was associated with improved survival. The four miRNAs that shared the lowest FDR were miR-320a, miR-361-5p, miR-21-5p and miR-103a-3p.

**Table 1:** List of overlapping miRNAs that correlated with breast cancer survival in TCGA.

Overlapping miRNA	Linear Model miRNA-Survival			Generalized Linear Model miRNA-Survival		
	P-Value	False Discovery Rate (FDR <0.05)	Coefficient (Estimate)	P-Value	False Discovery Rate (FDR <0.05)	Coefficient (Estimate)
hsa-miR-320a	0.00018	0.034	1.72E-05	0.00021	0.021	4.06E-04
hsa-miR-361-5p	0.00124	0.034	4.02E-05	0.00095	0.021	9.07E-04
hsa-miR-21-5p	0.00089	0.034	3.52E-08	0.00109	0.021	7.37E-07
hsa-miR-103a-3p	0.00151	0.034	3.16E-07	0.00105	0.021	7.92E-06
hsa-miR-374b-5p	0.00160	0.034	1.76E-04	0.00183	0.042	3.33E-03
hsa-miR-140-3p	0.00471	0.041	5.24E-06	0.00090	0.021	2.31E-04
hsa-miR-25-3p	0.00179	0.041	9.00E-07	0.00165	0.042	1.87E-05
hsa-miR-651-5p	0.00525	0.041	1.809E-03	0.00227	0.042	5.41E-02
hsa-miR-200c-3p	0.00271	0.041	6.97E-07	0.00290	0.044	1.40E-05
hsa-miR-30a-5p	0.00395	0.041	9.94E-08	0.00360	0.044	2.56E-06
hsa-miR-30c-5p	0.00362	0.041	1.05E-05	0.00383	0.044	2.03E-04
hsa-let-7i-5p	0.00544	0.041	2.09E-05	0.00424	0.044	4.29E-04

**Fig 2:**
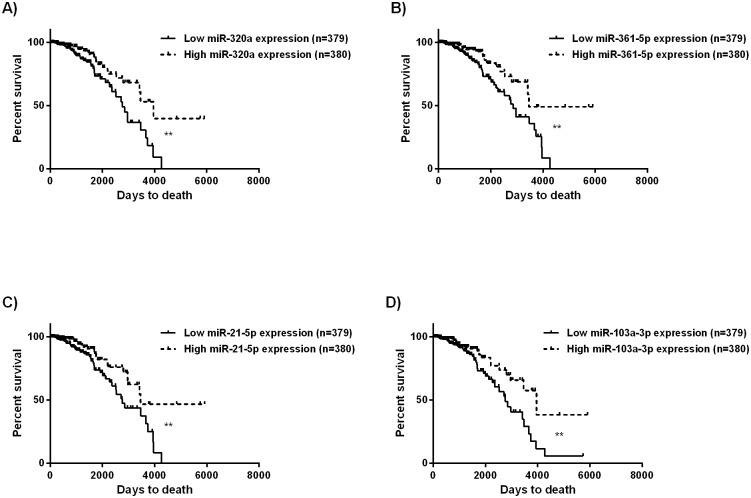
Kaplan-Meier survival plots for (A) miR-320a, (B) miR-361-5p, (C) miR-21-5p and (D) miR-103a-3p. The overall survival of BRCA patients was used for the survival analysis. Expression values of a miRNA were dichotomized into low and high expression using the median value of each specific miRNA. The solid line represents the low expression group and the dashed line represents the high expression group. The symbol ** signifies a log rank P-value < 0.05.

To further explore the clinical contributions of miR-320a, miR-361-5p, miR-21-5p, and miR-103a-3p to breast cancer survival, we generated Kaplan-Meier curves for each of the four miRNAs. Relatively high levels of miR-320a, miR-361-5p, miR-21-5p and miR-103a-3p were significantly associated with longer overall survival (for miR-320a, P = 0.0026; for miR-361-5p, P = 0.0021; for miR-21-5p, P = 0.0048; for miR-103a-3p, P = 0.0054). The Kaplan—Meier plots for miR-320a, miR-361-5p, miR-21-5p and miR-103a-3p are presented in Fig 2.

### Analysis of potential miRNA-gene interactions for miR-320a, miR-361-5p, miR-21-5p and miR-103a-3p through TCGA global gene expression

To explore the underlying biology of the observed miRNA-survival relationships in breast cancer, we then evaluated the potential gene targets of each of the four miRNAs. As described in the methods, two different versions of gene expression data (HiSeq V1 and V2) were utilized. Analysis completed by the TCGA Batch Effects Tool developed by MD Anderson Cancer Center revealed that both the V1 (DSC = 0.222, P<0.0005) and V2 (DSC = 0.273, P<0.0005) datasets had DSC < 0.5, indicating that batch effects in the gene expression data were weak. We performed separate linear regressions between each of the miRNAs of interest and the two gene expression datasets. The process of identifying the gene targets of the miRNAs can be found in Fig 1B.

After filtering for FDR<0.20, the negatively correlated miRNA-gene relationships for each of the 4 miRNAs were overlapped between those generated using the RNA-Seq V1 and V2 datasets. This yielded the following relationships: miR-320a retained 152 miRNA-gene relationships; miR-361-5p retained 66 miRNA-gene relationships; miR-21-5p retained 387, and miR-103a-3p retained 62 miRNA-gene relationships.

### Employing miRNA target prediction tools to pinpoint the possible gene targets of miR-320a, miR-361-5p, miR-21-5p and miR-103a-3p in breast cancer

A miRNA-gene interaction prediction tool, miRDB, was accessed to further narrow down the potential gene targets of each miRNA. miRDB yielded 581 targets for miR-320a; 340 targets for miR-361-5p; 240 targets for miR-21-5p; and 433 targets for miR-103a-3p. The overlap of these predicted targets and our TCGA gene expression analysis yielded 5 genes for miR-320a, 4 genes for miR-361-5p, 9 genes for miR-21-5p, and 1 gene for miR-103a-3p (S1 Table, along with relevant literature for each of the genes highlighted).

These final potential target genes were then evaluated for their association with breast cancer survival. We expected the genes to possess negative correlations with survival, acting as potential oncogenes that the miRNAs target in breast cancer. Expression of all of the genes except for *NCEH1* and *SYNGR2* possessed negative directionalities to survival, and 8 of the negative correlations were statistically significant (p<0.05) in at least the V1 or V2 gene expression dataset (Table 2). As predicted targets of miR-320a, *RAD51* and *RRP1B* were significantly correlated with worse patient survival in both expression profiles, while *TDG* was significantly correlated in the V2 dataset. A predicted target of miR-361-5p, *ARCN1* possessed a significant negative correlation to survival in the V2 gene expression profile. Of the 9 genes that miR-21-5p potentially targets in breast cancer, *MSH2* and *RMND5A* were significantly correlated with worse survival in both datasets, while *STAG2* and *UBE2D3* were significant in the V2 dataset. All eight miRNA-gene pairs identified by miRDB were evaluated using additional miRNA predictions tools: TargetScan and DIANA. We found every miRNA-gene pair to be corroborated by at least TargetScan and/or DIANA, in addition to the original miRDB prediction.

**Table 2:** Gene targets of the four miRNAs that correlate with breast cancer patient survival.

miRNA of Interest	Gene	miRNA-Gene				Gene-Survival	
		V1 Expression P-Values	V1 Coefficient (Estimate)	V2 Expression P-Values	V2 Coefficient (Estimate)	V1 Expression P-Values	V2 Expression P-Values
miR-320a	*RAD51*	0.00099	-99.1897	3.35E-07	-0.91591	0.02285	0.00077
	*RRP1B*	0.00262	-56.0061	6.55E-07	-0.21012	0.06015	0.00071
	*TDG*	0.00183	-61.8748	7.25E-08	-0.52252	0.24221	0.00979
miR-361-5p	*ARCN1*	0.00054	-6.68539	7.72E-13	-0.03011	0.13597	0.00247
miR-21-5p	*MSH2*	0.00032	-11808.4	1.88E-12	-112.194	0.04184	0.00070
	*RMND5A*	0.00182	-6872.72	6.80E-14	-79.7111	0.04832	0.00025
	*STAG2*	0.00300	-15179.1	1.93E-14	-65.7826	0.06023	0.00048
	*UBE2D3*	0.00390	-3272.45	2.03E-07	-29.326	0.13203	0.01033

## Discussion

The aim of this study was to discover breast cancer-associated miRNAs that significantly correlate with patient survival, and to propose the targets of the selected miRNAs within breast tumors. We accomplished this goal by employing a bioinformatics approach to one of the largest collections of molecular and clinical data for breast cancer via TCGA. Through our analysis, we propose that miR-320a, miR-361-5p, miR-21-5p may contribute to breast cancer survival by the negative regulation of breast oncogenes.

The initial correlation analysis between breast cancer survival and miRNA expression yielded 12 significant miRNAs: miR-320a, miR-361-5p, miR-103a-3p, miR-21-5p, miR-374b-5p, miR-140-3p, miR-25-3p, miR-651-5p, miR-200c-3p, miR-30a-5p, miR-30c-5p, and let-7i-5p. We focused our subsequent analysis on four of the most significant miRNAs; however, five of the other miRNAs identified have been suggested in breast cancer literature to possess tumor-suppressing properties, supporting the method we employed for discovery. One study performed in triple negative breast cancer study found that high levels of miR-374b-5p correlate with favorable outcomes and that miR-374-5p expression suppresses cell invasion *in vitro*. [13] Expression of miR-140 is found to be increasingly downregulated in breast cancer pathogenesis and progression, and miR-140 has been described to target the Wnt and *SOX* stem cell pathways to modulate breast cancer stem cell formation. [14–16] Low expression of miR-200c has been found to be associated with poor survival, and that upregulation of miR-200c inhibits cell proliferation and modulates cancer stem cell behavior. [17–20] miR-30a is a putative tumor suppressor that has been shown to negatively regulate processes such as cell proliferation and the epithelial-mesenchymal transition in breast cancer. [21–25] Lastly, higher miR-30c is associated with improved tamoxifen response in ER+ advanced breast cancers, and has been demonstrated to target cytoskeleton genes that are involved with cell invasion. [26, 27] In addition to confirming that these miRNAs appear to exert a protective phenotype in breast cancer, we undertook an in-depth investigation of the most significant miRNAs from our analysis—miR-320a, miR-361-5p, miR-21-5p, and miR-103a-3p—to identify clinically relevant gene targets.

The result that miR-320a is associated with tumor-suppression is consistent with both breast cancer literature and literature from other cancers. The miRNA has been demonstrated to inhibit breast cancer metastasis and invasion [28, 29] while also sensitizing breast cancer cells to chemotherapy [30, 31]. Additionally, miR-320a has been observed to be an independent prognostic factor where decreased miR-320a expression is correlated with lower survival in invasive breast cancer. [32] The anti-proliferative and tumor-suppressing effects of miR-320a have also been recorded in lung cancer [33], prostate cancer [34], gastric cancer [35], leukemia [36, 37], and nasopharyngeal carcinoma [38].

Our finding that higher miR-361 expression is a significant prognostic factor for improved survival is among the first in breast cancer. One breast cancer study had found that miR-361 was overexpressed in PARP1-upregulating *BRCA*-germline mutated and sporadic breast cancers. [39]. Another breast cancer study that examined 28 breast cancer samples observed that miR-361 upregulation was indicative of metastatic breast cancer, but an association with metastasis does not imply a statistically significant association with prognosis. [40] In addition, miR-361 has been found to be downregulated in hepatocellular carcinoma, and its expression has been shown to suppress cell proliferation and migration in hepatocellular, colorectal, gastric, and prostate cancer. [41–43]

Surprisingly, our TCGA analysis found that miR-21 was associated with improved breast cancer survival. This miRNA is widely regarded as an oncomiR in cancer literature. Upregulation of miR-21-5p has been associated with breast cancer pathogenesis, invasion, and metastasis. [44–50] Additionally, miR-21 overexpression has been found to promote breast cancer proliferation *in vitro*. [51] Upon examination of 344 primary breast cancer patients, Qian et al. reported that high miR-21 expression was associated with features of aggressive disease. However, they found no association between patient survival and miR-21 expression among all patients [52]. Based on this and other studies, we speculated that miR-21 is indeed an oncomiR. However, survival outcomes are a combined result of disease progression and response to treatment. It is plausible that the breast cancer patients who had more aggressive disease (as indicated by elevated miR-21 expression) were perhaps more responsive to certain treatments. Unfortunately, given the sparse treatment information in TCGA, this hypothesis is difficult to be assessed. Nonetheless, one needs to take into consideration that prognosis predictors may not directly relate to disease aggressiveness.

The literature for the role of miR-103 in cancer favors that miR-103 acts as an oncomiR rather than a tumor suppressor. High expression of miR-103 in relation to breast cancer has been correlated with metastasis, tumor relapse, and poor outcome. [53–56] On the other hand, one breast cancer study has found that miR-103 inhibits cancer stem cell formation in triple negative breast cancer. [57]

Of the five genes miR-320a was found to target in breast tumors, *RAD51*, *TDG*, and *RRP1B* are associated with poor patient survival. *RAD51* plays a central role in DNA repair by forming a complex with *BRCA2*, but its overexpression in breast cancer has been found to be associated with poor prognosis. [58, 59] In breast cancer cell lines, overexpression of *RAD51* has been shown to drive genomic instability and tumorigenesis as excess *RAD51* actually hampers the ability of cells to repair DNA. [60] Given *RAD51*’s oncogenic role, the targeting of *RAD5*1 as a cancer therapy is currently being explored for difficult-to-treat cancers such as triple negative breast cancer. [61, 62] *TDG* is a base excision repair enzyme that is believed to protect CpG islands from aberrant DNA methylation and to promote the demethylation of enhancers and promoters. [63] Interestingly, we observed that higher expression of *TDG* correlated with poorer breast cancer survival even though loss of *TDG* has been proposed to be involved in multiple myeloma [63, 64], pancreatic adenocarcinoma [63, 65], and rectal cancer [66]. *RRP1B* is another gene that significantly correlated with poor survival in TCGA, yet the gene is believed to interact with metastasis modifier genes to induce tumor suppression. [67, 68] It is important to note that the expression relationship of genes with cancer incidence cannot be translated directly to be equivalent to cancer prognosis. Our findings for *TDG* and *RRP1B* indicate that the two genes may possess unknown *in vivo* interactions that are associated with improved survival.

One of the four potential gene targets of miR-361-5p in breast cancer, *ARCN1* was found in our HiSeq V2 gene expression analysis to be significantly correlated with poor patient survival. The gene has been highlighted as a key determinant for sensitivity to the glycolytic inhibitor 2-deoxyglucose (2DG) in cancer cells, in which *ARCN1* knockdown sensitized cells to 2DG. [69] Although not statistically significant (p = 0.22 in V2), *ELL3* also possessed a negative correlation with breast cancer survival. Indeed, *ELL3* has been shown to increase cell proliferation, induce chemoresistance, and increase cancer stem cell populations, potentially through the MEK-extracellular signal-regulated kinase signaling pathway. [70]

The increased expression of the four genes *UBE2D3*, *RMND5A*, *STAG2*, and *MSH2*, which were predicted to be targets of miR-21-5p, all correlated with poorer survival. While *UBE2D3* has been described to be an *in vitro* tumor suppressor for its role in modulating radiosensitivity [71, 72] and proliferation [73], the *in vivo* data of TCGA suggested that higher *UBE2D3* expression predicts poorer clinical outcomes. One study has shown that the targeting of *RMND5A* significantly attenuates HeLa cell migration. [74] *STAG2* contributes to the cohesion complex and has been proposed as a prognostic biomarker for bladder and pancreatic cancer. [75–77] *MSH2* appears to play a complex role in breast cancer biology in which expression of the gene can be associated with tumor suppression [78, 79] or oncogenesis [80–82] depending on the context. One possible explanation is that *MSH2* may be downregulated as a breast cancer becomes invasive, but then *MSH2* expression becomes associated with breast cancer progression as the continued proliferation of tumor cells requires increased DNA mismatch repair. [83]

In summary, we have identified several miRNAs that are related to the survival of breast cancer. We propose that the miRNAs that relate to better prognosis may exert a protective phenotype by the silencing of breast oncogenes. Further research and validation experiments to explore the clinical and biological roles of miR-320a, miR-361-5p, miR-21-5p and miR-103a-3p may yield a better understanding of the mechanisms underlying breast cancer growth, metastasis, and survival.

## Supporting Information

S1 Table List of gene targets predicted for miR-320a, miR-361-5p, miR-21-5p and miR-103a-3p. Bolded font indicates that the gene was significantly correlated with breast cancer patient survival.
(PDF)
