# Supplementary material for: Identification of MicroRNAs as Breast Cancer Prognosis Markers through the Cancer Genome Atlas
Source: PLoS One. 2016 Dec 13;11(12):e0168284. doi: 10.1371/journal.pone.0168284 (PMC5154569; doi:10.1371/journal.pone.0168284)
Supplement: S1 Table — Bolded font indicates that the gene was significantly correlated with breast cancer patient survival. (PDF) [file pone.0168284.s001.pdf]

List of gene targets predicted for miR-320a, miR-361-5p, miR-21-5p and miR-103a-3p.

miRNA of Interest	Potential Gene Targets	Alternative Name of Gene	Relevant Literature (With Emphasis on Cancer)	P-values for miRNA-gene (<0.05 Threshold)
miR-361-5p	***ARCN1***	**Archain 1**	**In a screen to identify genes that control 2-deoxyglucose (2DG) sensitivity, *ARCN1* knockdown was found to sensitize cells to the glycolytic inhibitor. [1]**	**HiSeq_V1 P_1= 0.000544 HiSeq_V2 P_2= 7.72E-13**
	CREG1	Cellular Repressor of E1A stimulated Genes 1	There is conflicting literature for the role of *CREG1* in cancer. *CREG1* has been found to be overexpressed in non-small cell lung carcinoma cell lines with *KRAS* mutations and has also been found to be upregulated in gastric cancer tissues. [2,3] However, *CREG1* has also been found to be involved in cell senescence, to reduce cell proliferation, and to promote differentiation. [4-6]	P_1= 0.002726 P_2= 4.39E-07
	ELL3	Elongation Factor For RNA Polymerase II 3	In the breast cancer cell line MCF-7, *ELL3* expression has been found to promote cell proliferation and to increase cancer stem cell populations. [7] On the other hand, *ELL3* has been implicated in the stabilization process of p53, which ultimately results in increased cell apoptosis. [8]	P_1= 0.002395 P_2= 1.51E-07
	NCEH1	Arylacetamide Deacetylase-Like 1	*NCEH1* plays an important role in lipid metabolism and has been found to be overexpressed in numerous invasive cancer cell lines. [9-12] Knockdown of *NCEH1* in prostate cancer cells results in reduced cell migration, invasion, and survival. [13]	P_1= 0.002849782 P_2= 2.01E-08
miR-320a	*GSG2*	Germ Cell Associated 2 (Haspin)	*GSG2* plays a critical role in cell mitosis and has been found to be overexpressed in a number of neoplasms. *GSG2* has been suggested to be a potential therapeutic target for cancer. [14-16]	HiSeq_V1 P_1= 0.003303893 HiSeq_V2 P_2= 3.71E-06
	RAD51	**RAD51 Recombinase**	**In breast cancer, *RAD51* has been found to be overexpressed and to be associated with poor prognosis. [17] Another study has also shown that *RAD51* drives genomic instability in multiple breast cancer cell lines. [18] Additionally, downregulation of *RAD51* is associated with increased chemo-sensitivity. [19-20]**	**P_1= 0.000993014 P_2= 3.35E-07**
	RRP1B	**Ribosomal RNA Processing 1B**	**In one study, *RRP1B* was found to interact with metastasis modifier gene *SIPA1* to**	**P_1= 0.002623317 P_2= 6.55E-07**

			**regulate tumor suppressor genes. [21] *RRP1B* has also been found to interact with splicing regulator *SRSF1* to repress metastasis. [22]**	
	SYNGR2	Synaptogyrin 2	Expression of *SYNGR2* was included in a six-gene signature for thyroid tumors that could differentially diagnose malignant tumors and benign tumors. [23]	P_1= 0.000122781<br>P_2= 0.026343543
	TDG	**Thymine DNA Glycosylase**	**Deletion of *TDG* along with *PMS2* alterations contributes to a supermutator phenotype in both breast cancer and rectal cancer. [24-25]**	**P_1= 0.001831776<br>P_2= 7.25E-08**
miR-21-5p	*ATXN10*	Ataxin 10	This gene has been found to be elevated in human cachectic cancer patients, and inducing *ATXN10* in cardiomyocytes proved to be sufficient in producing cachexia phenotypes. [26] On the other hand, it has also been found that *ATXN10* is associated with cell senescence in human fibroblasts, and that knockdown of *ATXN10* promoted cell senescence avoidance. [27]	HiSeq_V1<br>P_1= 0.002806202<br>HiSeq_V2<br>P_2= 7.05E-09
	GATAD2B	GATA Zinc Finger Domain Containing 2B	In human fibroblasts, combinatorial knockdown of *GATAD2B* along with *ELAVL2* and *TEAD1* produced CD105+ cell populations, demonstrating increased differentiation. [28] Another study found that the stabilization of *GATAD2B* from *LRRC42* induction helps to promote cell growth in lung cancer cells. [29]	P_1= 0.002524675<br>P_2= 2.19E-10
	MSH2	**MutS Homolog 2**	***MSH2* appears to play multiple roles in breast cancer, producing research suggesting that *MSH2* possesses a dual role as oncogene and tumor suppressor depending on the context. *MSH2* expression has been observed to have increased expression in breast cancer tissues compared to normal tissues, and has been observed to have a negative correlation with histological grade. [30, 31] In contrast, *MSH2* has also been reported to be a tumor suppressor for its role in the TGF- β pathway. [32, 33]**	**P_1= 0.000321851<br>P_2= 1.88E-12**
	NKIRAS1	NFKB Inhibitor Interacting Ras-Like 1	In breast cancer and non-small cell lung cancer, *NKIRAS1* upregulation has been associated with the methylation of regulatory genes, potentially contributing to the	P_1= 0.004336416<br>P_2= 9.59E-10

			dysregulation of cell processes. [34, 35] However, *NKIRAS* has been found to be deleted or methylated in renal cancer. [36, 37]	
	PELI1	Pellino E3 Ubiquitin Protein Ligase 1	In leukemia, the constitutive expression of *PELI1* results in the development of lymphoid tumors. [38]	P_1= 0.001309759 P_2= 6.90E-06
	RMND5A	**Required For Meiotic Nuclear Division 5 Homolog A**	**In HeLa cells, the targeting of *RMND5A* through miR-138 dramatically reduces cell migration. [39] On the other hand, *RMND5A* expression has been found to increase after paclitaxel and carboplatin treatment for leukocyte gene expression. [40]**	**P_1= 0.001820747 P_2= 6.80E-14**
	STAG2	**Stromal Antigen 2**	***STAG2* is believed to play a tumor suppressing role for its function in the cohesion complex for both leukemia and pancreatic cancer. [41, 42] However, complete loss of *STAG2* in bladder cancer predicts good prognosis. [43]**	**P_1= 0.003000074 P_2= 1.93E-14**
	UBE2D3	**Ubiquitin Conjugating Enzyme E2 D3**	***UBE2D3* appears to act as a tumor suppressor in breast cancer because knockdown of *UBE2D3* in breast cancer cells augments cell proliferation and invasion. [44, 45] Additionally, inhibition of *UBE2D3* leads to radio-resistance. [44, 46]**	**P_1= 0.003901447 P_2= 2.03E-07**
	USP15	Ubiquitin Specific Peptidase 15	*USP15*'s role in enhancing TGF- β signaling has been found to be significant in glioblastomas, ovarian, and breast cancer. [47, 48] It has also been highlighted the *USP15* stabilizes MDM2, a negative regulator of p53, in cancer cells. [49]	P_1= 0.003470684 P_2= 3.27E-08
miR-103a-3p	*AMMECR1*	Alport Syndrome, Mental Retardation, Midface Hypoplasia And Elliptocytosis Chromosomal Region Gene 1	In ER+ breast cancer cells, miR-26 overexpression was found to inhibit estrogen-stimulated cell proliferation and tumor growth; *AMMECR1* was highlighted as a potential target of miR-26 as an estrogen responsive gene. [50]	HiSeq_V1 P_1= 0.004118655 HiSeq_V2 P_2= 2.55E-13

References

1. Kobayashi H, Nishimura H, Matsumoto K, Yoshida M. Identification of the determinants of 2-deoxyglucose sensitivity in cancer cells by shRNA library screening. Biochem Biophys Res Commun. 2015 Nov 6;467(1):121–7.
2. Clark DJ, Mei Y, Sun S, Zhang H, Yang AJ, Mao L. Glycoproteomic Approach Identifies KRAS as a Positive Regulator of CREG1 in Non-small Cell Lung Cancer Cells. Theranostics. 2016;6(1):65–77.
3. Xu L, Wang F, Liu H, Xu X-F, Mo W-H, Xia Y-J, et al. Increased expression of cellular repressor of E1A-stimulated gene (CREG) in gastric cancer patients: a mechanism of proliferation and metastasis in cancer. Dig Dis Sci. 2011 Jun;56(6):1645–55.
4. Moolmuang B, Tainsky MA. CREG1 enhances p16(INK4a) -induced cellular senescence. Cell Cycle. 2011 Feb 1;10(3):518–30.
5. Veal E, Groisman R, Eisenstein M, Gill G. The secreted glycoprotein CREG enhances differentiation of NTERA-2 human embryonal carcinoma cells. Oncogene. 2000 Apr 20;19(17):2120–8.
6. Veal E, Eisenstein M, Tseng ZH, Gill G. A cellular repressor of E1A-stimulated genes that inhibits activation by E2F. Mol Cell Biol. 1998 Sep;18(9):5032–41.
7. Ahn H-J, Kim G, Park K-S. Ell3 stimulates proliferation, drug resistance, and cancer stem cell properties of breast cancer cells via a MEK/ERK-dependent signaling pathway. Biochem Biophys Res Commun. 2013 Aug 9;437(4):557–64.
8. Ahn H-J, Kim K-S, Shin K-W, Lim K-H, Kim J-O, Lee J-Y, et al. Ell3 stabilizes p53 following CDDP treatment via its effects on ubiquitin-dependent and -independent

proteasomal degradation pathways in breast cancer cells. Oncotarget. 2015 Dec 29;6(42):44523–37.

9. Nomura DK, Durkin KA, Chiang KP, Quistad GB, Cravatt BF, Casida JE. Serine hydrolase KIAA1363: toxicological and structural features with emphasis on organophosphate interactions. Chem Res Toxicol. 2006 Sep;19(9):1142–50.

10. Jessani N, Liu Y, Humphrey M, Cravatt BF. Enzyme activity profiles of the secreted and membrane proteome that depict cancer cell invasiveness. Proc Natl Acad Sci U S A. 2002 Aug 6;99(16):10335–40.

11. Chiang KP, Niessen S, Saghatelian A, Cravatt BF. An enzyme that regulates ether lipid signaling pathways in cancer annotated by multidimensional profiling. Chem Biol. 2006 Oct;13(10):1041–50.

12. Shreder KR, Lin ECK, Wu J, Cajica J, Amantea CM, Hu Y, et al. Synthesis and structure-activity relationship of (1-halo-2-naphthyl) carbamate-based inhibitors of KIAA1363 (NCEH1/AADACL1). Bioorg Med Chem Lett. 2012 Sep 1;22(17):5748–51.

13. Chang JW, Nomura DK, Cravatt BF. A potent and selective inhibitor of KIAA1363/AADACL1 that impairs prostate cancer pathogenesis. Chem Biol. 2011 Apr 22;18(4):476–84.

14. Higgins JMG. Haspin: a newly discovered regulator of mitotic chromosome behavior. Chromosoma. 2010 Apr;119(2):137–47.

15. Cuny GD, Ulyanova NP, Patnaik D, Liu J-F, Lin X, Auerbach K, et al. Structure-activity relationship study of beta-carboline derivatives as haspin kinase inhibitors. Bioorg Med Chem Lett. 2012 Mar 1;22(5):2015–9.

16. Cuny GD, Robin M, Ulyanova NP, Patnaik D, Pique V, Casano G, et al. Structure-activity

relationship study of acridine analogs as haspin and DYRK2 kinase inhibitors. Bioorg Med Chem Lett. 2010 Jun 15;20(12):3491–4.

17. Le Scodan R, Cizeron-Clairac G, Fourme E, Meseure D, Vacher S, Spyratos F, et al. DNA repair gene expression and risk of locoregional relapse in breast cancer patients. Int J Radiat Oncol Biol Phys. 2010 Oct 1;78(2):328–36.

18. Parplys AC, Seelbach JI, Becker S, Behr M, Wrona A, Jend C, et al. High levels of RAD51 perturb DNA replication elongation and cause unscheduled origin firing due to impaired CHK1 activation. Cell Cycle. 2015;14(19):3190–202.

19. Huang F, Mazin A V. A small molecule inhibitor of human RAD51 potentiates breast cancer cell killing by therapeutic agents in mouse xenografts. PLoS One. 2014;9(6):e100993.

20. Hong K-J, Hsu M-C, Hung W-C. RECK impedes DNA repair by inhibiting the erbB/JAB1/Rad51 signaling axis and enhances chemosensitivity of breast cancer cells. Am J Cancer Res. e-Century Publishing Corporation; 2015;5(8):2422–30.

21. Nanchari SR, Cingeetham A, Meka P, Damineni S, Tipirisetti N, Padala C, et al. Rrp1B gene polymorphism (1307T>C) in metastatic progression of breast cancer. Tumour Biol. 2015 Feb;36(2):615–21.

22. Lee M, Dworkin AM, Gildea D, Trivedi NS, Moorhead GB, Crawford NPS. RRP1B is a metastasis modifier that regulates the expression of alternative mRNA isoforms through interactions with SRSF1. Oncogene. Nature Publishing Group; 2014 Apr 3;33(14):1818–27.

23. Rosen J, He M, Umbricht C, Alexander HR, Dackiw APB, Zeiger MA, et al. A six-gene model for differentiating benign from malignant thyroid tumors on the basis of gene

expression. Surgery. 2005 Dec;138(6):1050-6-7.

24. Vasovcak P, Krepelova A, Menigatti M, Puchmajerova A, Skapa P, Augustinakova A, et al. Unique mutational profile associated with a loss of TDG expression in the rectal cancer of a patient with a constitutional PMS2 deficiency. DNA Repair (Amst). 2012 Jul 1;11(7):616–23.

25. Dalton SR, Bellacosa A. DNA demethylation by TDG. Epigenomics. NIH Public Access; 2012 Aug;4(4):459–67.

26. Schäfer M, Oeing CU, Rohm M, Baysal-Temel E, Lehmann LH, Bauer R, et al. Ataxin-10 is part of a cachexokine cocktail triggering cardiac metabolic dysfunction in cancer cachexia. Mol Metab. Elsevier; 2016 Feb;5(2):67–78.

27. Rovillain E, Mansfield L, Lord CJ, Ashworth A, Jat PS. An RNA interference screen for identifying downstream effectors of the p53 and pRB tumour suppressor pathways involved in senescence. BMC Genomics. BioMed Central; 2011;12:355.

28. Ishihara Y, Tsuno S, Kuwamoto S, Yamashita T, Endo Y, Hasegawa J, et al. Hsa-miR-520d converts fibroblasts into CD105+ populations. Drugs R D. 2014 Dec;14(4):253–64.

29. Fujitomo T, Daigo Y, Matsuda K, Ueda K, Nakamura Y. Identification of a nuclear protein, LRRC42, involved in lung carcinogenesis. Int J Oncol. 2014 Jul;45(1):147–56.

30. Lira D, Teodoro TR, Pinhal MA da S, Fonseca FLA, Gehrke F de S, Azzalis LA, et al. Profile of hMSH2 expression in breast tumors and lymph nodes: a preliminary study. Eur Rev Med Pharmacol Sci. 2015 Sep;19(17):3229–33.

31. Chintamani, Jha BP, Bhandari V, Bansal A, Saxena S, Bhatnagar D. The expression of mismatched repair genes and their correlation with clinicopathological parameters and response to neo-adjuvant chemotherapy in breast cancer. Int Semin Surg Oncol. 2007;4:5.

32. Liu L, Zhou W, Cheng C-T, Ren X, Somlo G, Fong MY, et al. TGFβ induces "BRCAness" and sensitivity to PARP inhibition in breast cancer by regulating DNA-repair genes. Mol Cancer Res. 2014 Nov;12(11):1597–609.

33. Chen L, Li Y, Fu Y, Peng J, Mo M-H, Stamatakos M, et al. Role of deregulated microRNAs in breast cancer progression using FFPE tissue. PLoS One. 2013;8(1):e54213.

34. Pronina I V, Loginov VI, Burdennyy AM, Fridman M V, Kazubskaya TP, Dmitriev AA, et al. Expression and DNA methylation alterations of seven cancer-associated 3p genes and their predicted regulator miRNAs (miR-129-2, miR-9-1) in breast and ovarian cancers. Gene. 2016 Jan 15;576(1 Pt 3):483–91.

35. Braga EA, Loginov VI, Pronina I V, Khodyrev DS, Rykov S V, Burdennyy AM, et al. Upregulation of RHOA and NKIRAS1 genes in lung tumors is associated with loss of their methylation as well as with methylation of regulatory miRNA genes. Biochem Biokhimii͡a. 2015 Apr;80(4):483–94.

36. Gerashchenko G V, Bogatyrova OO, Rudenko EE, Kondratov AG, Gordiyuk V V, Zgonnyk YM, et al. Genetic and epigenetic changes of NKIRAS1 gene in human renal cell carcinomas. Exp Oncol. 2010 Jul;32(2):71–5.

37. Dmitriev AA, Rudenko EE, Kudryavtseva A V, Krasnov GS, Gordiyuk V V, Melnikova N V, et al. Epigenetic alterations of chromosome 3 revealed by NotI-microarrays in clear cell renal cell carcinoma. Biomed Res Int. 2014;2014:735292.

38. Park H-Y, Go H, Song HR, Kim S, Ha G-H, Jeon Y-K, et al. Pellino 1 promotes lymphomagenesis by deregulating BCL6 polyubiquitination. J Clin Invest. 2014 Nov;124(11):4976–88.

39. Li J, Chen Y, Qin X, Wen J, Ding H, Xia W, et al. MiR-138 downregulates miRNA

processing in HeLa cells by targeting RMND5A and decreasing Exportin-5 stability. Nucleic Acids Res. Oxford University Press; 2014 Jan;42(1):458–74.

40. González-Fernández R, Morales M, Avila J, Martín-Vasallo P. Changes in leukocyte gene expression profiles induced by antineoplastic chemotherapy. Oncol Lett. Spandidos Publications; 2012 Jun;3(6):1341–9.

41. Mullenders J, Aranda-Orgilles B, Lhoumaud P, Keller M, Pae J, Wang K, et al. Cohesin loss alters adult hematopoietic stem cell homeostasis, leading to myeloproliferative neoplasms. J Exp Med. 2015 Oct 19;212(11):1833–50.

42. Evers L, Perez-Mancera PA, Lenkiewicz E, Tang N, Aust D, Knösel T, et al. STAG2 is a clinically relevant tumor suppressor in pancreatic ductal adenocarcinoma. Genome Med. 2014;6(1):9.

43. Qiao Y, Zhu X, Li A, Yang S, Zhang J. Complete loss of STAG2 expression is an indicator of good prognosis in patients with bladder cancer. Tumour Biol. 2016 Aug;37(8):10279–86.

44. Wang W, Yang L, Hu L, Li F, Ren L, Yu H, et al. Inhibition of UBE2D3 expression attenuates radiosensitivity of MCF-7 human breast cancer cells by increasing hTERT expression and activity. PLoS One. 2013;8(5):e64660.

45. Mittal MK, Singh K, Misra S, Chaudhuri G. SLUG-induced elevation of D1 cyclin in breast cancer cells through the inhibition of its ubiquitination. J Biol Chem. 2011 Jan 7;286(1):469–79.

46. Yang H, Wu L, Ke S, Wang W, Yang L, Gao X, et al. Downregulation of Ubiquitin-conjugating Enzyme UBE2D3 Promotes Telomere Maintenance and Radioresistance of Eca-109 Human Esophageal Carcinoma Cells. J Cancer. 2016;7(9):1152–62.

47. Eichhorn PJA, Rodón L, Gonzàlez-Juncà A, Dirac A, Gili M, Martínez-Sáez E, et al. USP15 stabilizes TGF-β receptor I and promotes oncogenesis through the activation of TGF-β signaling in glioblastoma. Nat Med. 2012 Mar;18(3):429–35.

48. Zhang L, Zhou F, García de Vinuesa A, de Kruijf EM, Mesker WE, Hui L, et al. TRAF4 promotes TGF-β receptor signaling and drives breast cancer metastasis. Mol Cell. 2013 Sep 12;51(5):559–72.

49. Zou Q, Jin J, Hu H, Li HS, Romano S, Xiao Y, et al. USP15 stabilizes MDM2 to mediate cancer-cell survival and inhibit antitumor T cell responses. Nat Immunol. 2014 Jun;15(6):562–70.

50. Tan S, Ding K, Li R, Zhang W, Li G, Kong X, et al. Identification of miR-26 as a key mediator of estrogen stimulated cell proliferation by targeting CHD1, GREB1 and KPNA2. Breast Cancer Res. 2014;16(2):R40.
